# Prospects for lithium treated patients with severe renal impairment

**DOI:** 10.1186/s40345-025-00372-z

**Published:** 2025-02-14

**Authors:** Harald Aiff, Per-Ola Attman, Mihaela Golic, Bernd Ramsauer, Staffan Schön, Steinn Steingrimsson, Jan Svedlund

**Affiliations:** 1 Department of Psychiatry and Neurochemistry, Institute of Neuroscience and Physiology, Sahlgrenska Academy, University of Gothenburg, Göteborg, Sweden; 2 Psykiatri Affektiva, Department of Psychiatry, Region Västra Götaland, Gothenburg, Sweden; 3 Department of Psychiatry, Region Halland, Varberg, Sweden; 4 Department of Nephrology, Skaraborg Hospital, Skövde, Sweden; 5 Swedish Renal Registry, Jönköping County Hospital, Jönköping, Sweden

## Abstract

**Objectives:**

To study the prospects for lithium treated patients who develop end stage renal disease (ESRD) and the role of renal replacement therapy (RRT).

**Methods:**

Retrospective analysis of survival, somatic comorbidity, lithium treatment and eligibility for renal replacement therapy in adult patients with at least one eGFR < 30 ml/min/1.73 m^2^. Subjects were selected from our laboratory database (s-Lithium and s-creatinine) from 1980 to 2017.

**Results:**

620 (14%) of 4396 patients with a lithium history had at least one measurement of eGFR < 30 ml/min/1.73 m^2^. 302 (49%) patients had a transient decrease in renal function with subsequent improvement, 135 (22%) patients died with acute renal failure, while 153 (25%) developed chronic kidney disease stage 4 (CKD4) and 33 (5%) required RRT. RRT-treated patients represent only a fraction of the total ESRD population. Median survival time from the debut of CKD4 was 13.9 years in patients < 65 years and 4.4 years in older patients.

100 of the 153 patients with CKD4 continued lithium treatment. There was no significant difference in survival after the debut of CKD4 between the patients who stopped lithium treatment and those who continued.

**Conclusions:**

A measurement of eGFR < 30 ml/min/1.73 m^2^ reflects a significant loss of renal function. In half of the patients it was due to a transient functional disturbance without long-term consequences. A quarter of patients had acute renal failure and died within days while the remaining quarter progressed to CKD4. Despite irreversible renal damage, patient survival can be counted in several years after debut of renal insufficiency with appropriate care including RRT. As the treating psychiatrist, it is important to consult with nephrology when renal function starts to deteriorate, to optimise somatic treatment.

## Introduction

Lithium treatment is the recommended long term prophylactic treatment by most guidelines for bipolar disorder and an alternative for recurrent and treatment resistant depression (McIntyre et al. 2020). Long-term treatment with lithium carries a risk for a number of adverse effects, notably serious nephropathy (Davis et al. 2018). The magnitude of the risk is still a matter of controversy and varies between different studies (Aiff et al. 2015). It has even been questioned if lithium treatment carries any additional risk compared to other treatments (Bosi et al. 2023; Kessing et al. 2015; Aiff et al. 2014; Kessing et al. 2024).

It is advised that lithium treatment should be conducted according to guidelines that include regular monitoring of renal function i.e. serum creatinine concentration to estimate glomerular filtration rate (eGFR) (Golic et al. 2018).

A significant reduction of eGFR may indicate either a transient disturbance of renal hemodynamics or is one of the first signs of a chronic kidney disease (CKD). It is usually the treating psychiatrist who is first faced with the interpretation of the finding and the subsequent questions i.e. the possibly gloomy prospects for the patient to eventually face renal failure and need for renal replacement therapy (RRT), and if lithium treatment therefore should be stopped. The current literature provides little guidance to the psychiatrist faced with these questions.

Discontinuation of lithium therapy does not invariably result in a slower progression of renal impairment. Some authors suggest that there is a “point of no return” where discontinuation of lithium below a certain eGFR level does not stop the progression to renal failure. If such a point exists is still a matter of discussion. A Dutch study (Hoekstra et al. 2022) followed patients with eGFR < 60 ml/min/1.73 m2 who discontinued lithium. Patients with higher eGFR in the group appeared to benefit more from discontinuing lithium, than those with lower eGFR. In addition, Rej et al. (2013) followed older patients with eGFR < 60 ml/min/1.73 m^2^ for 60 months and observed a trend of creatinine increase in patients who continued compared to those who discontinued lithium treatment. On the contrary, a Danish register study found that continuing lithium after a diagnosis of CKD does not result in an increased risk of RRT (Kessing et al. 2017). The variability in outcomes complicates the decision-making process for psychiatrists, as discontinuation of lithium therapy often results in recurrence of manic or depressive episodes, with uncertain benefits for renal health. The present study aims at describing the prospects for lithium treated patients who already developed severely decreased renal function and the role of renal replacement therapy.

## Methods

### Data collection

The database at the Department of Clinical Chemistry at Sahlgrenska University Hospital, Gothenburg, Sweden was used as the main data source. It includes data from laboratories serving the public hospitals and out-patient clinics in the greater Gothenburg area encompassing approximately 10% of the Swedish population. All patients with one positive lithium measurement between 1 January 1980 and 31 December 2017 were included in the study. We retrieved the age and sex of these patients together with all serum lithium and creatinine concentration levels.

Relevant clinical information was obtained from structured reviews of individual medical charts, the National Cause of Death Register (The Swedish National Board for Health and Welfare. 2024), the Swedish Renal Register (SNR 2023) and the Swedish Death Index (2023). 

Based on the clinical information the structured review focused on somatic comorbidity including cause of death, consultation with nephrology and decision to start RRT. The obtained information was then further categorised for tabular presentation. We defined somatic comorbidity as the presence of chronic diseases that needed hospital treatment or long-term pharmacological treatment. The present study includes cardiovascular disease as a composite characterisation (hypertension, angina pectoris, myocardial infarction, peripheral vascular disease, stroke), diabetes mellitus, malignancy, urological conditions and pre- or coexisting renal disease.

### Laboratory methods

Before 1 June 2004 serum creatinine concentrations were determined by a picrate method. Thereafter, a more specific enzymatic method was used and previous values were adjusted as described elsewhere (Aiff et al. 2015). Serum lithium concentrations were determined by flame photometry.

### Exclusions

Patients younger than 18 years were excluded. Patients without creatinine measurements might have had their follow-up elsewhere and were excluded due to missing data.

### Definitions

The glomerular filtration rate was estimated (eGFR) from the serum creatinine concentration, age and sex according to the Revised Lund-Malmö formula devised by Björk et al. (2011). The eGFR was used to categorise the level of renal function in 5 stages of chronic kidney disease (CKD) according to the KDIGO guidelines (Stevens et al. 2024). This staging of CKD identifies patients with an eGFR < 30 ml/min/1.73 m^2^ consistently for 90 days or more as having CKD stage 4 (CKD4) and an eGFR < 15 ml/min/1.73 m^2^ as CKD stage 5 (CKD5) and ESRD. The present study is focused on patients with severely decreased renal function, defined as eGFR < 30 ml/min/1.73 m^2^ registered after having at least one previous measurable plasma lithium concentration.

To qualify for CKD4 stage in this study, we required at least two measurements of eGFR < 30 ml/min/1.73 m^2^ with at least 90 days separation and with no subsequent eGFR ≥ 30 ml/min/1.73 m^2^. The CKD4 date was defined as the first non-improving eGFR < 30 ml/min/1.73 m^2^. If a patient had only one measurement of eGFR < 30 ml/min/1.73 m^2^ OR measurements of eGFR ≥ 30 ml/min/1.73 m^2^ after eGFR < 30 ml/min/1.73 m^2^, this was regarded as “No CKD4”. CKD4-patients with at least one following measurement of eGFR < 15 ml/min/1.73 m^2^, were considered as CKD5 and ESRD.

We defined patients who had at least one measurable lithium concentration on or after the date for CKD4 as continuing lithium treatment, and patients without a lithium measurement as stopping lithium treatment.

### Patients

The database included 4396 patients with at least one measurable lithium concentration and serum creatinine concentration during the study period. Of them, 3776 patients had no measurement of eGFR < 30 ml/min/1.73m^2^ during the study period or no lithium measurement prior to eGFR < 30 ml/min/1.73m^2^ and were excluded from further analysis.

### Statistical analysis

Laboratory data was processed in Excel (Microsoft Corporation, Redmond, WA, USA), and additional variables were calculated. Patient charts were reviewed for additional data. The data was analysed using custom Python (ver 3.11.5) scripts using Pandas (The pandas development team 2024) in the Spyder IDE (ver 5.4.3) (Raybaut, 2024). The Kaplan–Meier diagrams, median survival time, log-rank testing and the Cox Proportional Hazard ratio calculations were calculated using the lifelines package (ver 0.28.0) Davidson-Pilon 2024.

### Ethics approval and consent to participate

The study received approval from the Regional Ethical Review Board in Gothenburg 2015-08-15 with completion 2018-05-08, Dnr: 594-15. Patient consent was waived. The study adhered to the principles outlined in the Declaration of Helsinki, as revised in 1989.

### Funding declaration

This study was funded by Psykiatri Affektiva, Sahlgrenska University Hospital, Sweden.

## Results

### Study patients

**Fig. 1:**
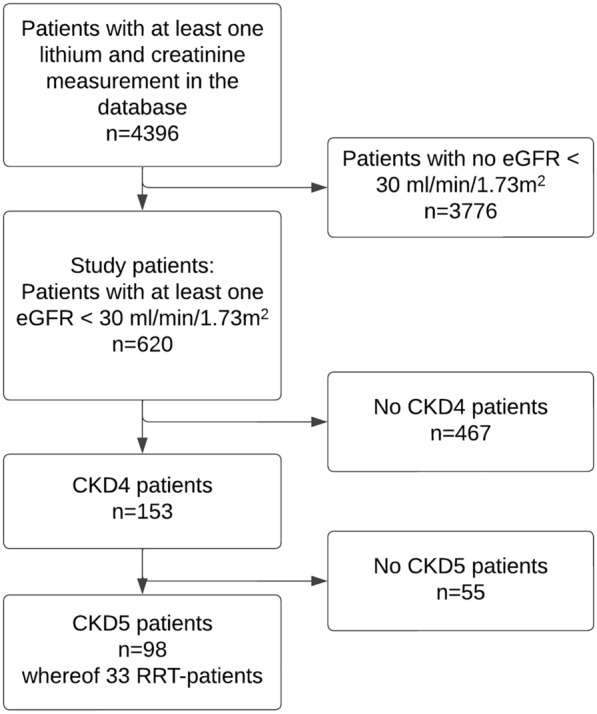
Flow chart of patient selection

620 patients (388 female and 232 male) had at least one measurement of eGFR < 30 ml/min/1.73 m^2^ during the study period and constituted the study group. 467 patients never reached CKD4 as defined above. They fall into three categories: patients with transient renal insufficiency (n = 302), patients with acute renal failure (n = 135) and patients lost to follow-up (n = 30). See Fig. 1.

#### No CKD4 patients

##### Patients with transient renal insufficiency

302 patients in the study group (49%) had a transient episode of renal functional impairment with eGFR < 30 ml/min/1.73 m^2^ during a median of 2 days with varying degrees of subsequent renal functional improvement and therefore never reached CKD4. Their recovered final eGFR in the database is listed in Table 1:

**Table 1:** Patients with transient episodes of renal functional impairment (eGFR < 30 ml/min/1.73 m2)

	No	Dead by end of study	Median time (years) between first eGFR < 30 and last observation (min–max)	Age at first eGFR < 30 median (min–max)	Age at death median (min–max)	Recovered eGFR interval in ml/min/1.73 m2			
						(> 90)	(90–60)	(59–45)	(44–30)
All patients	302	174	3 (0–25)	72 (28–99)	79 (43–102)	15	79	84	124
Women	187	108	3 (0–25)	73 (28–99)	81 (45–102)	10	44	50	83
Men	115	66	2 (0–25)	69 (29–92)	77 (43–94)	5	35	34	41

##### Patients with acute renal failure

135 patients (74 female and 61 male) in the study group (22%) died within 90 days (median 4 days) from first eGFR < 30 ml/min/1.73 m^2^, with irreversible loss of renal function, at a median age of 79 years (range: 30–101). The underlying cause for loss of renal function was predominantly related to fatal hemodynamic imbalance from cardiac failure, postoperative or septic shock, dehydration, metastatic malignancy or final stages of other somatic illness.

##### Patients without follow up

17 patients with only one eGFR < 30 ml/min/1.73 m^2^ and no further eGFR measurements were alive on December 31, 2017. Another 13 patients died later than 90 days from first eGFR < 30 ml/min/1.73 m^2^ but without follow up measurements in the database.

#### CKD4 patients

**Fig. 2:**
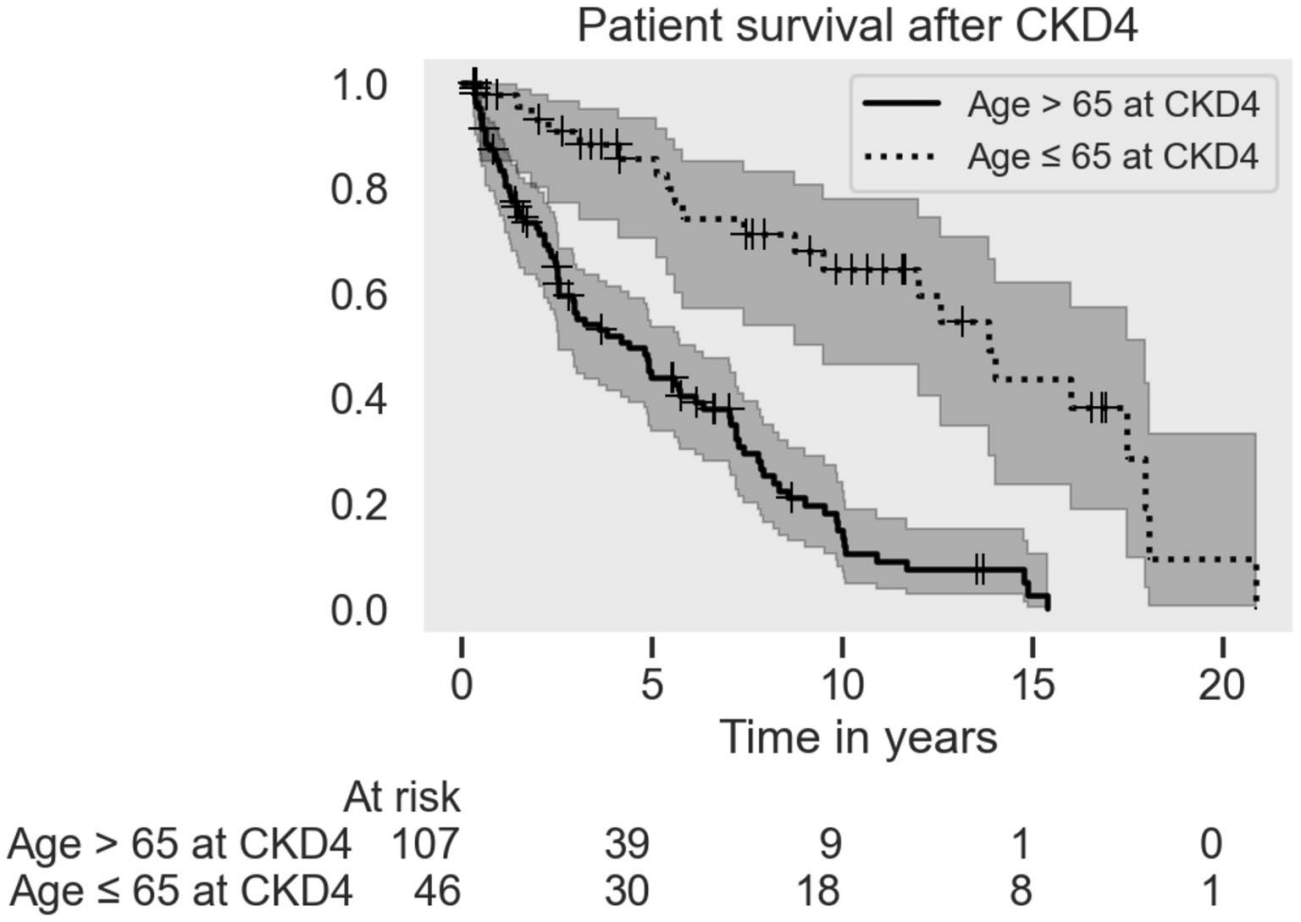
Patient survival from the debut of CKD4 status, grouped by age, with 95% confidence intervals. Includes patients who progressed to CKD5 and those subsequently treated with RRT. The difference in survival between the two groups is significant (p < 0.05)

153 patients in the study group (25%) fulfilled our criteria for CKD4 as defined above at a median age of 72 (range: 42–95) years. Patient survival after CKD4 debut was highly correlated with age, each year increasing the risk of death with hazard ratio of 1.08 (95%CI 1.06–1.11, p < 0.05). The median survival was 13.9 years for patients younger than 65 years and 4.4 years for patients older than 65 years. Median age at CKD4 was 73 for women and 71 for men. Median survival time was 7.0 years for women and 5.1 years for men. See Fig. 2.

77% of the 153 patients had somatic comorbidity (Table 2). 32 patients died with CKD4 without progression to CKD5 after a median of 1.3 years at an age of 82 (range: 69–97) years. The causes of death were dominated by cardiovascular disease and malignancies (Table 3). 23 patients were alive with CKD4 by the end of the observation period.

**Table 2:** Somatic comorbidities in CKD4 patients

	All patients	Female patients	Male patients
No of patients	153	103	50
Age at CKD4 status (median)	72	73	71
No of patients with comorbidity	118 (77%)	81 (79%)	37 (74%)
No of patients with cardiovascular disease	80 (52%)	54 (52%)	26 (52%)
No of patients with malignancy	34 (22%)	22 (21%)	12 (24%)
No of patients with primary kidney condition	26 (17%)	14 (14%)	12 (24%)
No of deaths	105	75	30

**Table 3:** Age at death and causes of death in the 105 CKD4 patients deceased by the end of study

	All patients	Female patients	Male patients
Age of death median, years	80	80	80
Cause of death			
Cardiovascular	25%	31%	10%
Malignancy	10%	8%	17%
Wasting/uremia	24%	24%	23%
Other	16%	13%	23%
Not known	25%	24%	27%

#### CKD5 patients

**Fig. 3:**
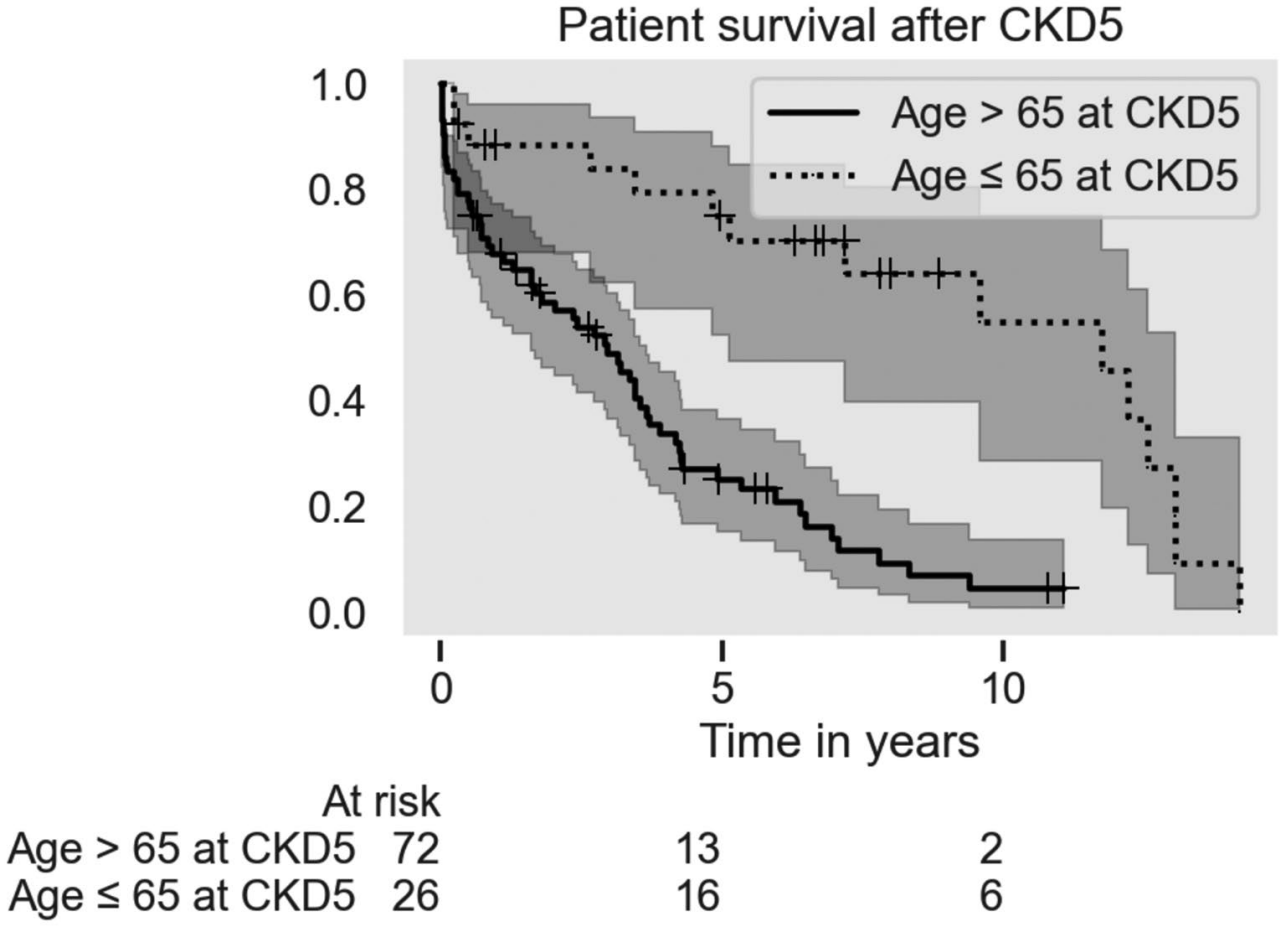
Patient survival from the debut of CKD5 status, grouped by age, with 95% confidence intervals. Patient survival after CKD5 debut separated by age

98 patients (68 female, 30 male) of the 153 CKD4 patients survived to reach ESRD with CKD5 after a median of 2.9 (range: 0–12.5) years at a median age of 73 (range: 46–98) years. 76 of the 98 patients had significant somatic comorbidity and the main causes of death were uremia, vascular disease, respiratory failure and malignancies. 33 patients were subsequently treated with RRT. 52 patients who were not treated with RRT died after a median of one year (0–12,6) from first eGFR < 15 ml/min/1.73 m^2^, at a median age of 82 (55–98) years. 13 patients were alive with CKD5 status at the end of study period. See Fig. 3.

Patient survival after the debut of CKD5 was highly dependent on age (HR = 1.06 per year, 95% CI 1.02–1.09, p < 0.05, Fig. 3) while the contribution of RRT was not significant (HR = 0.15, 95% p = 0.37). The median overall survival time was 11.7 years for patients younger than 65 years and 3.0 years for patients older than 65 years.

##### Patients treated with RRT

The structured review of medical records included evaluation of consultation with nephrologist when documented. Consultation for patients who did not receive RRT was documented in 36 out of 65 patients (55%). In 10 cases the kidney function at time was judged sufficient for medical treatment alone. In 26 cases the patient was considered unable to benefit from RRT because of somatic comorbidity (SNR 2023), inability to cooperate (Socialstyrelsen. 2024) or because of the patient's decision (McIntyre et al. 2020). In 29 cases there was no specific documentation of nephrological consultation.

In total 33 patients (21 female and 12 male) were treated with RRT. One patient was primarily treated with renal transplantation while all other patients commenced RRT with either hemodialysis or peritoneal dialysis with subsequent renal transplantation in five cases.

Female patients started RRT at a median age of 69 years and died at a median age of 74 years. Male patients started RRT younger at a median age of 63 years and died at a median age of 66 years.

Twenty-four patients had significant somatic comorbidity; notably vascular disease (n = 17), malignancy (n = 6) and diabetes mellitus (n = 8). Twenty-one patients died during the study period. The main causes of death were vascular disease and uremia after cessation of RRT due to wasting and dementia.

##### Lithium treatment

One hundred patients were still treated with lithium at the time of CKD4-diagnosis. The median lithium concentration of the last obtained lithium value was 0.43 mmol/l in the group that stopped treatment before CKD4 and 0.52 mmol/l in the group that continued lithium treatment. Sixty-eight patients had lithium for more than one year after CKD4. The further decline of eGFR was not influenced by lithium cessation. The median survival time was 7.2 years for patients who continued treatment and 5.8 years for patients who stopped treatment. After adjusting for age (which is the major contributing factor) and sex, there was no significant difference in survival after the debut of CKD4 between patients without further lithium treatment and patients who continued lithium treatment. 13 of the 33 RRT patients continued their lithium treatment during RRT.

## Discussion

The rationale for this study was to illustrate the prospects for patients who experience severely decreased kidney function following periods of lithium treatment. The results of the study were based on multiple unselected measurements of serum creatinine concentrations from nearly five thousand lithium-treated patients during more than three decades and shows that about 15% of lithium-treated patients will over time experience a major reduction of their renal function either transient or chronic. The study therefore aimed at providing insight in the clinical significance of loss of at least half of the renal function which is far more than expected from age alone and thus of concern for the individual patient and the physician when prescribing life-long prophylactic treatment.

We included all patients in the database with documented use of lithium at any time during the study period thus encompassing both short and long-term lithium users. This naturalistic cohort included both patients with lithium nephropathy and patients with collateral causes for renal damage. We also focused on patients who presented with an estimated glomerular filtration rate of less than 30 ml/min/1.73 m^2^ which reflects such a severe disturbance of renal function that cannot be accounted for by age related pathophysiology.

An elevated serum creatinine concentration reflects only the glomerular filtration rate during the preceding days and rapid changes in renal hemodynamics may result in seemingly dramatic loss of filtration capacity that can be restored ad integrum within short. Long-term lithium treatment frequently results in nephrogenic diabetes insipidus and increased vulnerability to fluid loss with hypovolemia, hypotension and reduced renal perfusion (Bendz et al. 1994; Tabibzadeh et al. 2022). In half of the studied cases the loss of renal function was transient and presumably related to disturbances of renal hemodynamics and of salt-water balance. Correction of the hydrodynamic imbalance generally leads to restoration of renal filtration capacity without long-term deterioration of function and lithium treatment can be continued.

However, in a number of cases the sudden and irreversible loss of renal function was a consequence of rapid development of serious somatic comorbidity leading to acute renal failure and patient death within days or weeks, generally at an advanced age. In these cases the fatal outcome was caused by the comorbidity without relation to lithium treatment or its indication.

In contrast to the above patients with rapid development of fatal renal insufficiency a large number of the patients with a debut of renal insufficiency will subsequently continue to lose filtration capacity at a varying pace. With consistent eGFR < 30 ml/min/1.73 m^2^ the patient is regarded by the KDIGO definition as having permanent and severe renal function loss (CKD4). The further deterioration of the renal function is often slow.

Not unexpectedly, the dominating major prognostic factor for patient survival after CKD4 debut was patient age. Our results nevertheless show that a significant number of older patients with CKD4 survive for several years and usually without incapacitating symptoms of uremia. One third of the deceased patients died at an advanced age with CKD4 but without ESRD and mainly from cardiovascular disease and malignancies which is comparable to non-lithium-related CKD4 populations (Ramspek et al. 2022; Zhang et al. 2009).

Only a minority of CKD4 patients were below 65 years old at time of CKD4 status. These patients live significantly longer than their older peers, long enough to experience a further deterioration of renal function. They are likely to become high consumers of nephrology services and should be noticed in earlier stages of renal decline.

Patients in the present study were to a great extent burdened with somatic comorbidity, notably vascular disease and diabetes mellitus, which in turn may be aggravated by the renal insufficiency. Cardiovascular comorbidity has also been identified as a strong risk factor for loss of renal function in lithium-treated patients (Aiff et al. 2019). Close attention to treatment of somatic comorbidity is therefore mandatory in the care of the patients with compromised renal function.

In our catchment area, there were more female than male patients treated with lithium which was reflected in the study population (The Swedish National Board of Health and Welfare. 2024). Female patients suffered their loss of renal function at an older age and had longer survival than male patients. This is in line with the observation that renal disease in female patients has a slower rate of progression compared to male patients, possibly related to incidence of hypertension (Neugarten and Golestaneh 2013; Chesnaye et al. 2021).

About two-thirds of the patients who entered CKD4 status experienced a further deterioration of renal function to CKD5 with ESRD. We found that also at this advanced stage the progression of renal failure to terminal uremia can be remarkably slow with patients surviving for more than a year from the debut of CKD5, reaching a median age of 80 years. When the residual renal function drops below 15 ml/min/1.73 m^2^, the renal failure is progressively manifest with prominent clinical symptoms which can be managed with conservative measures to an extent, and with subsequent RRT when indicated.

One third of the patients who developed ESRD were treated with RRT, mainly dialysis, and in a few cases with renal transplantation. RRT was initiated when the foregoing therapeutic measures could no longer control the manifestations of uremia in patients judged to benefit from RRT. Chronic RRT implies a physically demanding treatment and patient cooperation. The access to medical records enabled us to explore the underlying conditions for choice of treatment modality including nephrological consultation in 70% of the cases.

The results show that a considerable number of the CKD5 patients had advanced comorbidity, judged to constitute inability to benefit from RRT. The patients for whom we lacked information about consultation with a nephrologist died at an advanced age when RRT represents a very demanding treatment which makes it likely the reason why consultation for RRT was not contemplated. The majority of our patients reached ESRD during the second millennium when access to RRT was no longer limited by treatment facilities. Patients started RRT at the same average age as the general RRT population in Sweden during the study period (SNR 2023).

Interestingly, more than half of the patients who developed CKD4 were still on lithium treatment and most of them continued their treatment, often until death or during subsequent RRT. Our results do not support that cessation of lithium treatment after CKD4 debut influences further patient survival, with the reservation that they may be subject to selection bias. This is in line with a Danish population study, where cessation of lithium in patients with CKD was not associated with less RRT (Kessing et al. 2017). This finding together with the risk of exacerbation of the underlying affective disorder if lithium is discontinued suggests keeping lithium in most cases.

Besides monitoring the lithium treatment strictly according to the guidelines, the psychiatrist should also interpret the laboratory results dynamically and in conjunction with the last couple of years’ results, paying attention to “creeping creatinine”, i.e. steady increase of the creatinine value over time. The threshold for contacting a nephrologist should be lower for younger patients.

One strength of the present study is that patients were identified by measurement of their individual renal function and its development over time. This approach reveals that assessment of incidence of renal failure based on RRT register numbers only catches a fraction of the patients with ESRD thus underestimating the true incidence of ESRD (Kessing et al. 2015). In our data, only one third of the patients with ESRD would be identified from our RRT-register. This seems to be comparable with the incidence of RRT in a Swedish CKD population where many of the older patients with advanced CKD never received RRT (Lundström et al. 2017).

### Limitations

This is a retrospective register study with added chart reviews. The data was not originally collected for this purpose which accounts for the unavoidable incompleteness inherent in the study. As described elsewhere, the influx of data was more sparse during the first decade of the study period, limiting the information on debut of renal insufficiency during that period (Golic et al. 2018). Patients moving out from the catchment area before the end of study were lost to follow-up but could have developed CKD4 or CKD5 without our knowledge.

The evaluation of nephrology consultation and referral of patients for RRT relies on completeness of medical records which was more limited in patients who developed renal failure during the first decade of the study period. The findings in this respect are therefore more representative of the later decades of the study period.

## Conclusions

A marked reduction of renal function with eGFR < 30 ml/min/1.73 m^2^ is a potentially serious prognostic finding. In half of the patients it was a sign of transient and treatable loss of renal function without long-term consequences. In a further quarter of the patients, this finding resulted from an acute renal failure with rapid patient death. For the last quarter of patients it was a process developing over several years, with further progression of renal damage. In our study population, patient survival at this stage was not dependent on whether lithium treatment was continued or not. Using creatinine based estimations of renal function over time, this study reveals that RRT register data substantially undervalues the true incidence of ESRD.

However, despite the irreversible renal damage, patient survival can be counted in several years after debut of renal insufficiency with appropriate care, maintained lithium treatment, and including RRT when indicated.

Cessation of lithium treatment following a CKD4 diagnosis was not the rule in our study population. In several cases, it appears that continuing treatment despite renal damage was considered the most appropriate action.

As the treating psychiatrist, it is essential to collaborate with the nephrologist to optimize the management of somatic comorbidities.

## Data Availability

The data supporting this study cannot be made publicly available due to lack of ethics committee permission, as data-sharing was not part of the approval process. The coding specifications are available from the corresponding author upon reasonable requests.
